# Effect of Inoculated Lactic Acid Fermentation on the Fermentable Saccharides and Polyols, Polyphenols and Antioxidant Activity Changes in Wheat Sourdough

**DOI:** 10.3390/molecules26144193

**Published:** 2021-07-10

**Authors:** Ewa Pejcz, Sabina Lachowicz-Wiśniewska, Paulina Nowicka, Agata Wojciechowicz-Budzisz, Radosław Spychaj, Zygmunt Gil

**Affiliations:** 1 Department of Fermentation and Cereals Technology, Wrocław University of Environmental and Life Sciences, 51-630 Wrocław, Poland; sabina.lachowicz@upwr.edu.pl (S.L.-W.); agata.wojciechowicz-budzisz@upwr.edu.pl (A.W.-B.); radoslaw.spychaj@upwr.edu.pl (R.S.); zygmunt.gil@upwr.edu.pl (Z.G.); 2 Department of Fruit, Vegetable and Nutraceutical Technology, Wrocław University of Environmental and Life Sciences, 51-630 Wrocław, Poland; paulina.nowicka@upwr.edu.pl

**Keywords:** sourdough fermentation, inoculation, lactic acid bacteria, FODMAP, fructans, antioxidant activity

## Abstract

Inoculation of sourdough allows the fermentation medium to be dominated by desired microorganisms, which enables determining the kinetics of the conversion of chemical compounds by individual microorganisms. This knowledge may allow the design of functional food products with health features dedicated to consumers with special needs. The aim of the study was to assess the dynamics of transformations of fermentable oligosaccharide, disaccharide, monosaccharide and polyol (FODMAP) compounds from wheat flour as well as their antioxidant activity during inoculated and spontaneous sourdough fermentation. The FODMAP content in grain products was determined by the fructan content with negligible amounts of sugars and polyols. To produce a low-FODMAP cereal product, the fermentation time is essential. The 72 h fermentation time of *L. plantarum*-inoculated sourdough reduced the FODMAP content by 91%. The sourdough fermentation time of at least 72 h also positively influenced the content of polyphenols and antioxidant activity, regardless of the type of fermentation. The inoculation of both *L. plantarum* and *L. casei* contributed to a similar degree to the reduction in FODMAP in sourdough compared to spontaneous fermentation.

## 1. Introduction

Sourdough is traditionally prepared by mixing flour with water, and subjecting this mixture to a multi-stage spontaneous fermentation, which is carried out by exogenous flour microflora, including mainly 10^4^–10^7^ CFU/g of bacteria and yeast [1]. In order to shorten the technological process and increase its repeatability, it is an increasingly common practice to add starter cultures to sourdough. A group of lactic acid bacteria (LAB) plays a key role in these processes and has a long and safe history of use and consumption in fermented foods and beverages [2]. Another solution is to inoculate fermented products, including bakery sourdoughs, with pure cultures of bacteria or yeast proliferated to a desired number of colony-forming units [3,4]. Sourdough fermentation allows the fermentation medium to be dominated by desired microorganisms, which enables determining the kinetics of the conversion of chemical compounds of flour by individual microorganisms, and their targeted selection [5].

Cereal products make up a significant proportion of food consumed by the worldwide population. Wheat bread is considered a rich source of fermentable oligosaccharides, disaccharides, monosaccharides and polyols (FODMAPs) due to a high content of fructans, formed by the aggregation of fructose molecules. FODMAPs are easily fermentable, highly osmotic carbohydrates, including fructooligosaccharides (FOSs), galactooligosaccharides (GOSs), lactose, fructose and polyols (notably sorbitol and mannitol) [6,7,8].

The effect of FODMAPs on human health is determined by the amount of sugar delivered to the body within food. The appropriate intake of FODMAPs has a positive impact on human health because certain FODMAP sugars exhibit prebiotic effects [9,10]. The excess intake of FODMAP-rich products (above 20 g/day) can lead to sugar accumulation in the intestines, which in turn may induce various gastric ailments, which are acute in people suffering from irritable bowel syndrome [6,8].

Irritable bowel syndrome (IBS) is a gastrointestinal disorder that can appear in persons of various ages, genders and ethnical origins. It affects 4–20% of the population. Its typical symptoms usually appear after the intake of FODMAP-containing food products and include abdominal discomfort and stomachache, accompanied by flatulence, constipation or diarrhea [11,12]. Simple sugars and polyols exhibit a stronger osmotic effect, whereas saccharides such as fructans, FOSs and GOSs are more susceptible to fermentation by the intestinal microbiome [8,13].

Research has shown that FODMAP components trigger clinical symptoms in IBS patients [14,15,16]. One of the diets most often recommended by dietitians to help combat IBS symptoms is the low-FODMAP diet. Its principle is to reduce the intake of food products containing short-chain carbohydrates, which are rapidly absorbable in the human gastrointestinal tract [17]. In addtion, dietitians advise paying attention to the fructose:glucose ratio in consumed food products and recommend that their levels are similar or a higher glucose content. This can help improve the intestine’s capability to absorb fructose [6]. Food products rich in these compounds include cereal products rich in fructans [11].

Fructans are not digested nor absorbed in the human digestive tract [6]. When ingested in small amounts, fructans have some health benefits but their excess can cause various ailments of the digestive tract [9]. The low-FODMAP dietary guidelines recommend substituting traditional bread with gluten-free products [11,14,18]. Wheat bakery products have significantly higher contents of protein, dietary fiber, minerals and vitamins than the gluten-free ones. Therefore, the exclusive consumption of gluten-free products can lead to deficiencies of these compounds in the body [19].

The FODMAP content in bread depends on both flour type and bread-making method [20]. The content in bread can be reduced in many ways, one of which is to use sourdough in the bread-making process. Another means is to appropriately select microorganisms responsible for the fermentation and degradation of sugars that trigger the gastrointestinal disorders [8]. In wheat bread, the above goal can also be achieved by extending fermentation time, which not only improves the flavor values of bread but also effectively decreases FODMAP content [20]. Fructans present in high quantities in cereal kernels can be degraded during sourdough fermentation. The consumption of sourdough bread has been proved to have a beneficial effect on mitigating irritable bowel syndrome symptoms [8]. In order to produce a low-FODMAP bread, LAB should also be added to the sourdough as they enhance the metabolic activity of fermenting flora. Apart from their capability to metabolize fructans, LAB can also convert free fructose to mannitol. In addition, they produce α-galactosidase, i.e., an enzyme responsible for breaking the bonds between the molecules of sugars constituting GOSs [8]. In turn, the enzymes capable of mannitol conversion are secreted by, e.g., *Lactobacillus delbrueckii*, *Lactobacillus casei*, *Lactobacillus plantarum* and *Lactobacillus salivarius* [7,21]. Bread produced with sourdough requires longer fermentation, which entails multiple changes in the carbohydrate composition. Microbial invertase rapidly degrades flour saccharose into glucose and fructose. Afterward, glucose is consumed as a source of energy, whereas fructose can be reduced by heterofermentative LAB to mannitol. All fermentable carbohydrates are rapidly depleted in the first hours of fermentation, whereas the carbohydrates featuring a high degree of polymerization (like fructans) are consumed later [8,22].

Sourdough fermentation used in bread making improves the nutritional value and antioxidative properties of bread, as well as its taste, aroma, texture and stability, and finally the bioaccessibility of its elements [23]. The antioxidant activity of the components of sourdough depends on the type of inoculum used for fermentation [24] and sourdough fermentation time [25]. The aim of the study was to assess the dynamics of transformations of FODMAP compounds from wheat flour as well as the antioxidant activity of nutrients of flour during inoculated and spontaneous sourdough fermentation.

## 2. Results and Discussion

### 2.1. Dynamics of pH Changes during Fermentation

Table 1 shows the results of the pH measurement of spontaneously fermented and lactobacilli-inoculated wheat sourdoughs. In each type of sourdough, the greatest decrease in pH was observed after the first 24 h of fermentation. During fermentation, LAB produce lactic acid, which results in a lower pH level [26]. In spontaneously fermenting and *L. casei*-inoculated sourdough after the first day of fermentation, pH remained at a similar level. A further slight decrease in pH was observed in *L. plantarum*-inoculated sourdough when the fermentation time was extended to 72 h. The study by Menezes et al. [8] also showed the greatest decrease in the pH level in the first hours of wheat dough fermentation, until relatively stable values were achieved after several stages. Fluctuations in the pH level affect the action of amylases. A study by Struyf et al. [27], showed that lowering the pH level has an effect on maltose release but has no effect on other saccharides.

### 2.2. Dynamics of FODMAP Content Change during Fermentation

Changes in the FODMAP content in the sourdoughs during their fermentation are presented in Table 2. Fructans constituted the majority of these compounds in the tested samples. The content of fructans in the sourdough was influenced by the fermentation time and the type of LAB used. Each extension of the fermentation time resulted in a significant decrease in the content of fructans in the sourdough compared to the control, which was non-fermented sourdough. For each of the sourdough types, the content of fructans decreased with the fermentation time and reached the lowest values after 72 h of fermentation. A similar relationship between the extension of the fermentation time and the decrease in the content of fructans was observed by Struyf et al. [28], where after 1 h of fermentation, more than half of the fructans were degraded in the dough compared to the content of fructans present in the flour. In the study by Gélinas et al. [29], it was found that 20% of fructans were degraded after the dough-mixing process. Then, by fermenting the dough with yeast for 180 min, the fructan content was reduced by 82% compared to the amount of fructans present after mixing the dough. For fermentation lasting 24 h, the sourdough fermented with *L. plantarum* achieved the lowest content of fructans among the analyzed sourdoughs. However, in the case of 48 h and 72 h fermentation, the lowest fructan content was observed in sourdoughs inoculated with *L. casei*. Fraberger et al. [30] tested 13 strains of microorganisms for their ability to reduce fructans and found that the metabolism of microflora contributed to a significant reduction in the content of fructans in the dough compared to the control sample. Sourdough fermented with *L. casei* bacteria reached a lower content of fructans faster compared to sourdough fermented with *L. plantarum* and this could be due to the higher activity of *L. casei* enzymes than *L. plantarum* [7].

The non-fermented sourdough control sample did not contain free glucose (Table 2). After 24 h of spontaneous fermentation, the glucose content was 0.06 g/100 g d.m., then after 48 h its value increased to 0.08 g/100 g d.m., and after 72 h it dropped back to 0. In the case of sourdough inoculated with *L. plantarum*, after 24 h and after 48 h of fermentation, the glucose content was 0.05 g/100 g, and after 72 h, its content in the sourdough decreased to 0. In the case of sourdough fermented with *L. casei*, the content of glucose increased to 0.2 g/100 g d.m. after 24 h of fermentation, and after both 48 and 72, its value dropped to 0. The glucose level in sourdough is determined by the content of damaged starch and the activity of β-amylase and amyloglucosidase [22]. It was also found that it is a factor blocking the transformations of, among others, sucrose, raffinose and mannitol. A fermentation time of 72 h led to a complete reduction of glucose in the sourdough. Further changes in glucose may result in the formation of CO_2_, lactate, acetate and ethanol [7,21,27]. No fructose content was observed in any of the analyzed sourdough. It is consumed quickly and can also be converted into mannitol by lactobacilli [7].

The presence of mannitol was not found in any of the analyzed sourdough during the first 24 h of fermentation, because mannitol is formed from the degradation of fructose, which is transformed in the later stages of fermentation [31]. No mannitol was detected in the spontaneously fermented sourdough for 24 as well as 48 h, and after 72 h its value increased to 0.007 g/100 g d.m. In the sourdough with the addition of *L. casei* bacteria, after 48 h, the mannitol content was found at the level of 0.006 g/100 g of dry matter, and after 72 h, the content decreased to 0.002 g/100 g d.m. In sourdough fermented with *L. plantarum*, the level of mannitol remained at 0 during 72 h of fermentation. Gänzle [21] claims that the degradation of mannitol requires the enzymes of lactobacilli found, among others, in *L. casei* bacteria. In the spontaneously fermented sourdough, mannitol was present only after 72 h of fermentation, which results from the metabolism of fructose. It is converted into mannitol by lactobacilli, therefore in pure bacterial cultures fructose was degraded to mannitol faster than in the case of spontaneously fermenting sourdough. Mannitol metabolism, however, may be inhibited by the presence of glucose [7,21].

The total FODMAP content before fermentation was 1.153% d.m. and was determined by the fructan content of the flour. The FODMAP content of wheat is influenced by its variety. Ziegler et al. [20] studied the content of compounds from the FODMAP group in two wheat flour varieties and showed that it is from 1.24 ± 0.38 to 2.01 ± 0.42 g/100 g d.m. The fermentation of the flour always resulted in a significant decrease in the FODMAP content, but with a different effect depending on the type of sourdough used and its duration. In the spontaneously fermenting sourdough, the FODMAP content decreased with the extension of the fermentation time, and it reached the lowest value after 72 h. The FODMAP content in the spontaneously fermenting sourdough in the study by Menezes et al. [8] was 0.553 g/100 g d.m. and 0.603 g/100 g d.m. depending on various parameters of sourdough fermentation. A similar effect was observed in *L. casei*-inoculated sourdough, but with slight difference in FODMAP content after 48 and 72 h of fermentation. Sourdough fermentation with the addition of *L. plantarum* resulted in the lowest FODMAP content after 72 h and was constant after 24 and 48 h. In the study of Menezes et al. [8], it is claimed that the sourdough biotechnology requires a longer fermentation time than is usually used in bread making (0.5–3 h). Carbohydrates such as sucrose, maltose, glucose and fructose are depleted quickly during the first hours of fermentation, while higher-polymerized carbohydrates such as fructans are used later, so longer fermentation of sourdough will degrade all FODMAP components more efficiently. Comparing sourdoughs after 24 h of fermentation, the one with the addition of *L. plantarum* had the lowest content of FODMAP, while after 48 and 72 h of fermentation, the lowest FODMAP concentration was in sourdough with *L. casei*. Finally, after 72 h of fermentation with the addition of *L. casei*, the lowest FODMAP level of 0.076 g/100 g d.m. was achieved, which is a reduction of their content by 93%. It is important to select the microorganisms responsible for the fermentation of the sourdough. Appropriate LAB have enzymes that degrade FODMAP components, and they also have the ability to lower the pH of the environment, thanks to which the activity of the enzymes increases, which leads to a reduction in the FODMAP content. By lowering the FODMAP content in wheat bread, it is possible to reduce the symptoms of irritable bowel syndrome [7,8,30].

### 2.3. Dynamics of Polyphenolic Compounds and Antioxidant Activity Changes during Fermentation

The total content of polyphenols and the antioxidant activity of sourdoughs are presented in Table 3. The content of polyphenols in the sourdough was higher after each type of fermentation than before. However, the content of polyphenols in the analyzed material did not totally change. The matrix of the components of flour and sourdough was loosened during fermentation and water-extractable polyphenols were released. The fermentation process may increase the antioxidant activity by increasing the amount of easily extractable phenolic compounds [24]. Spontaneously fermenting sourdough reached the highest content of polyphenols after 24 h of fermentation, after which their amount remained on a similar level. *L. casei*-inoculated sourdough contained the highest amounts of polyphenols after 48 and 72 h of fermentation. The content of polyphenols in *L. plantarum*-inoculated sourdough increased significantly after 24 h of fermentation and then again after 72 h. Chiș et al. [32] observed an increase in the content of polyphenols with the fermentation time with the addition of *L. plantarum*, which is explained by their proteolytic activity’s influence on the polyphenol profile. LAB can affect polyphenols, improving their solubility [33].

The antioxidant activity measured by both ABTS and FRAP methods of the spontaneously fermenting sourdough increased significantly after 24 h of fermentation and then after 72 h. In the study of Banu et al., 2010 [24], the addition of starter cultures containing *Lactobacillus rhamnosus* to the dough increased the antioxidant activity compared to spontaneous fermentation. In this study, the sourdough inoculated with *L. casei* showed a higher antioxidant activity against the ABTS radical after 24 h fermentation than before, and the highest value was achieved after 72 h of fermentation. A significant increase in the ability to reduce iron ions of this sourdough took place only after 72 h of fermentation. The antioxidant activity of *L. plantarum*-inoculated sourdough increased significantly after 72 h of fermentation. In the study of Banu et al. [24], antioxidant activity (measured with ABTS and DPPH methods) of 20 h spontaneously fermented dough was almost two times higher than before fermentation. Colosimo et al. [25] observed a significant increase in polyphenols and antioxidant activity with the fermentation time of the sourdough, which should last 72 h and preferably 96 h. In a study by Rodríguez et al. [34], *L. plantarum* was able to increase the antioxidant activity and improve the aroma profile of the product by degrading certain phenolic components through the metabolic activity of the LAB. The metabolic activity of LAB influences the levels of bioactive ingredients, which allows for an increase in antioxidant activity. During fermentation with their participation, antioxidant peptides are released, which increases the amount of phenols and antioxidant activity by acidification and hydrolysis of more complex and glycosylated forms [24,35]. Extending fermentation to 72 h resulted in an increase in the antioxidant activity of sourdoughs by 83 to 98% compared to the samples before fermentation, regardless of the type of sourdough fermentation. Sourdough fermentation can remove peptides associated with human intolerance to grain products. It can also lead to the production of bioactive peptides with antioxidant potential, which may affect the bioavailability of nutrients [25].

## 3. Materials and Methods

### 3.1. Material

Wheat flour type 650 was supplied from GoodMills (Stradunia, Poland). The flour particle size was 93 ± 0.3 μm, it had falling number of 390.5 ± 1.0 and contained 14.72 ± 0.02% protein (data not shown). Lyophilizates of two safe and well-described species of lactic acid bacteria: *Lactobacillus casei*, catalogue number 20,011 and *Lactobacillus plantarum*, catalogue number 20,174, were purchased from DSMZ—German Collection of Microorganisms and Cell Cultures (Leibniz, Germany).

Lactobacilli were grown in Man, Rogosa and Sharp medium (MRS) (Sigma-Aldrich, Hamburg, Germany) and incubated under aerobic conditions at 37 °C until the late exponential growth phase was reached (about 24 h). Cells were harvested by centrifugation at 10,000 rpm for 10 min at 4 °C. Dilutions were made in saline solution plated on MRS 273 agar, resulting in a concentration of about 10^9^ CFU/mL.

The next multiplication of microorganisms took place by preparing a mixture of 100 g of flour, 300 mL of water and 20 mL of liquid microorganism culture (*L. casei* and *L. plantarum*). A mixture without the addition of bacteria was prepared based on the spontaneous fermentation of microorganisms found naturally in the flour. The fermentation lasted three days at 28 °C.

Sourdoughs were made from a combination of flour (500 g), water (500 mL) and the appropriate liquid sourdough prepared in the previous step (50 mL). The fermentation of sourdoughs was carried out for 24, 48 and 72 h at a temperature of 28 °C.

### 3.2. Methods

#### 3.2.1. Dynamic of Fermentation

The pH of the sourdoughs was determined in four replicates after 24, 48 and 72 h of fermentation using the potentiometric method. The pH of the non-fermented sourdough was used as a control. The samples were frozen, freeze dried, ground and vacuum packed for further determinations.

#### 3.2.2. Determination of Fructans

The content of fructans in the freeze-dried sourdough samples was determined using the fructan determination kit based on AOAC Method 999.03 [36], which is based on the determination of the fructose content in the samples resulting from the enzymatic breakdown of fructans. Using a spectrophotometer, the fructose content was measured at a wavelength of λ = 410 nm. The determination was performed in duplicate.

#### 3.2.3. Determination of Sugar and Polyol Content by HPLC-ELSD

Preparation of samples for the determination of sugar and polyol content consisted of adding 10 g of the analyzed sample into a volumetric flask, filling the volumetric flask to 50 mL and boiling and shaking the samples in a boiling water bath for 20 min. Then, 100 mL of cooled samples was made up with distilled water, 10 mL of the extract was centrifuged (10,000 rpm, 10 min) and the samples were filtered on a Sep-Pak C-18.

The content of sugars and polyols was determined by the HPLC method coupled with a light scattering detector. A 40 µL sample was injected by an autosampler (L-7200) onto a Unison UK-Amino 3 µL (3 mm × 250 mm) column (Imtakt, Kyoto, Japan). Detection was performed using an evaporative light scattering detector (PL-ELS 1000) with the following input parameters: evaporator temperature −80 °C; nebulizer temperature −80 °C; nitrogen flow −1.2 SLM. The elution was performed at 30 °C in an isocratic flow using 85% acetonitrile solution at a flow rate of 0.7 mL/min. FODMAP content was identified by comparing with standard HPLC area measurements. The measurements were performed in duplicate and the results were expressed in grams/100 g dry weight of the product. The sum of the FODMAPs was calculated from the fructan content and those identified in the samples: fructose, mannitol and glucose.

#### 3.2.4. Determination of Polyphenolic Compounds and Antioxidant Activity

The extraction for the antioxidant capacity was conducted following a protocol described by Lachowicz et al. [37]. The total polyphenolic content of the sourdough samples was determined using the Folin–Ciocalteu spectrophotometric method [38]. The absorbance at 765 nm was measured after 1 h, using the UV-2401 PC spectrophotometer (Shimadzu, Kyoto, Japan). The results were expressed as mg of gallic acid equivalents (GAE) per 100 g of dry sourdough. Data were expressed as the mean value for three measurements. The ABTS and FRAP methods were carried out with the methods described by Re et al. [39] and Benzie and Strain [40]. The absorbance was measured at 734 nm and 593 nm using the UV-2401 PC spectrophotometer (Shimadzu, Kyoto, Japan). The results of antiradical capacity were expressed as Trolox equivalents in mmol per 100 g of dry sample. Data were expressed as the mean value for three measurements.

### 3.3. Statistic Analysis

The results were statistically analyzed with the Statistica 13.3 software package (StatSoft, Tulsa, OK, USA). One-way ANOVA at *p* ≤ 0.05 was calculated and homogeneous groups according to the Duncan test were estimated.

## 4. Conclusions

The FODMAP content in grain products turned out to be determined by the fructan content with negligible amounts of sugars and polyols. To produce a low-FODMAP cereal product, the fermentation time is essential, and its extension to 72 h or more allows for a strong reduction in the content of these compounds. A sourdough fermentation time of at least 72 h also positively influences the content of polyphenols and antioxidant activity, regardless of the type of fermentation. The inoculation of both *L. plantarum* and *L. casei* contributed to a similar degree to the reduction of FODMAPs in sourdough compared to spontaneous fermentation. Knowledge of the processes that take place during the fermentation of inoculated sourdoughs may allow the production of food products designed according to the needs of consumers.

## Figures and Tables

**Table 1:** pH of wheat sourdough during fermentation.

Fermentation Time [h]/Sourdough Type	Spontaneous Fermentation	*Lactobacillus casei*	*Lactobacillus plantarum*
0	6.159 a	6.159 a	6.159 a
24	3.410 c	3.592 b	3.566 b
48	3.441 b	3.506 c	3.437 c
72	3.410 c	3.593 b	3.416 d

Values represent the means of four replicates. Mean values in columns with different letters are significantly different according to Duncan test at *p* ≤ 0.05.

**Table 2:** The content of FODMAP components (g/100 g d.m.) in wheat sourdough.

Sourdough Type	Fermentation Time [h]	Fructan	Glucose	Fructose	Mannitol	Sum of FODMAPs
unfermented sourdough	0	1.15 a	0.00 e	nd	0.000 d	1.15 a
spontanous fermentation	24	0.42 b	0.06 c	nd	0.000 d	0.48 b
	48	0.28 d	0.08 b	nd	0.000 d	0.35 c
	72	0.18 e	0.00 e	nd	0.007 a	0.19 d
*Lactobacillus casei*	24	0.39 bc	0.20 a	nd	0.000 d	0.45 b
	48	0.11 ef	0.00 e	nd	0.006 b	0.12 de
	72	0.07 f	0.00 e	nd	0.002 c	0.08 e
*Lactobacillus plantarum*	24	0.31 cd	0.05 d	nd	0.000 d	0.36 c
	48	0.31 cd	0.05 d	nd	0.000 d	0.36 c
	72	0.10 ef	0.00 e	nd	0.000 d	0.10 de

Nd: not detected. Values represent the means of two replicates. Mean values in columns with different letters are significantly different according to Duncan test at *p* ≤ 0.05.

**Table 3:** The content of polyphenolic compounds and antioxidant activity of wheat sourdough.

Sourdough Type	Fermentation Time [h]	Polyphenolic Compounds [mg/100 g d.m.]	ABTS [mmol Trolox/100 g d.m.]	FRAP [mmol Trolox/100 g d.m.]
unfermented sourdough	0	208.30 c	1.95 c	0.96 c
spontaneous fermentation	24	273.74 ab	2.16 bc	1.36 b
	48	270.54 ab	2.18 bc	1.27 b
	72	262.35 b	3.88 a	1.80 a
*Lactobacillus casei*	24	263.13 b	2.48 bc	1.06 c
	48	270.65 ab	2.01 c	1.03 c
	72	295.96 ab	3.60 a	1.85 a
*Lactobacillus plantarum*	24	251.38 b	1.96 c	1.09 c
	48	260.07 b	1.91 c	1.11 c
	72	309.59 a	3.58 a	1.89 a

Values represent the means of three replicates. Mean values in columns with different letters are significantly different according to Duncan test at *p* ≤ 0.05.

## Data Availability

Results will be available from the corresponding author.

## Sample Availability

Sourdough lyophilisates are available from the authors upon reasonable request.
